# Genetic differentiation of Mexican Holstein cattle and its relationship with Canadian and U.S. Holsteins

**DOI:** 10.3389/fgene.2015.00007

**Published:** 2015-02-09

**Authors:** Adriana García-Ruiz, Felipe de J. Ruiz-López, Curtis P. Van Tassell, Hugo H. Montaldo, Heather J. Huson

**Affiliations:** ^1^Facultad de Estudios Superiores Cuautitlán, Universidad Nacional Autónoma de MéxicoAjuchitlán, Mexico; ^2^Centro Nacional de Investigación Disciplinaria en Fisiología y Mejoramiento Animal, Instituto Nacional de Investigaciones Forestales, Agrícolas y Pecuarias – Secretaría de Agricultura, Ganadería, Desarrollo Rural, Pesca y AlimentaciónAjuchitlán, Mexico; ^3^Animal Genomics and Improvement Laboratory, Agricultural Research Service, United State Department of AgricultureBeltsville, MD, USA; ^4^Department of Animal Science, Cornell UniversityIthaca, NY, USA

**Keywords:** genetic differentiation, Holstein, admixture, linkage disequilibrium

## Abstract

The Mexican Holstein (HO) industry has imported Canadian and US (CAN + USA) HO germplasm for use in two different production systems, the conventional (Conv) and the low income (Lowi) system. The objective of this work was to study the genetic composition and differentiation of the Mexican HO cattle, considering the production system in which they perform and their relationship with the Canadian and US HO populations. The analysis included information from 149, 303, and 173 unrelated or with unknown pedigree HO animals from the Conv, Lowi, and CAN + USA populations, respectively. Canadian and US Jersey (JE) and Brown Swiss (BS) genotypes (162 and 86, respectively) were used to determine if Mexican HOs were hybridized with either of these breeds. After quality control filtering, a total of 6,617 out of 6,836 single nucleotide polymorphism markers were used. To describe the genetic diversity across the populations, principal component (PC), admixture composition, and linkage disequilibrium (LD; *r^2^*) analyses were performed. Through the PC analysis, HO × JE and HO × BS crossbreeding was detected in the Lowi system. The Conv system appeared to be in between Lowi and CAN + USA populations. Admixture analysis differentiated between the genetic composition of the Conv and Lowi systems, and five ancestry groups associated to sire’s country of origin were identified. The minimum distance between markers to estimate a useful LD was found to be 54.5 kb for the Mexican HO populations. At this average distance, the persistence of phase across autosomes of Conv and Lowi systems was 0.94, for Conv and CAN + USA was 0.92 and for the Lowi and CAN + USA was 0.91. Results supported the flow of germplasm among populations being Conv a source for Lowi, and dependent on migration from CAN + USA. Mexican HO cattle in Conv and Lowi populations share common ancestry with CAN + USA but have different genetic signatures.

## INTRODUCTION

Dairy farms in Mexico are extremely heterogeneous (e.g., different herd sizes, feeding systems, reproductive management, etc.). Conventional (Conv) dairy farms have an average herd size of 230 head and are highly mechanized and milk yield is relatively high. Cows on these farms are typically grouped in pens and rations usually include high proportions of concentrates. Low income (Lowi) systems vary with region with sizes ranging from 3 to 30 cows, with animals usually spending part of the day grazing, but may also be housed in pens (Amendola, 2002). Additionally, Lowi farms rely heavily on the use of unpaid family labor and the typical herd is smaller than Conv herds. The Mexican Holstein (HO) population has depended on US and Canada genetics for some years (Powell and Wiggans, 1991). Recently, germplasm from European populations have been introduced, however, these populations also tend to be highly influenced by US and Canadian bulls (Norman and Powell, 1999). This information was expected in the Conv Mexican systems where pedigree information was available, but little was known about the genetic influence on the Lowi system because of incomplete or unavailable pedigree information.

Before single nucleotide polymorphism (SNP) data became available, genetic diversity and relatedness was studied through the analysis of pedigree information which, unfortunately, is not always complete. This incomplete data renders results inaccurate or limits the performance of studies of individuals or populations (Boichard et al., 1997). Currently, with the availability of SNP data, it is possible to estimate breed or population compositions without the previous knowledge of ancestry information (Sölkner et al., 2010; Frkonja et al., 2012). Different analyses based on genomic information have been used to study genetic diversity of populations. In this study, principal component analysis (PCA) was performed to describe the breed or geographic allele variation, whereas admixture analysis was used to describe the structure of populations. Persistence phase of linkage disequilibrium (LD) analysis allowed the characterization of the degree of agreement of LD across distances between populations.

Principal component analysis was first used in human populations to generate maps summarizing the allele frequency of different geographic areas (Cavalli-Sforza et al., 1994; Silva-Zolezzi et al., 2009) and more recently for understanding relationship among cattle breeds (Bovine HapMap Consortium, 2009; Lewis et al., 2011). Currently, PCA is used to control spurious genome wide association in populations structured with individuals of different geographic areas or even when geography does not explain the genetic background (Novembre and Stephens, 2008). In this study, PCA was used to identify differences among HO cattle originating from CAN + USA and two Mexican production systems.

Admixture is defined as the mixing of genomes of divergent parental origins (Buerkle and Lexer, 2008), which implies the presence of multiple genetically distinct groups or breeds in a population (Wang et al., 2005). Admixture can be studied at an individual (Tang et al., 2005) or population level (Buerkle and Lexer, 2008; Frkonja et al., 2012). These haplotype blocks vary in size because of the random nature of recombination, but become progressively shorter by further recombination with increasing generations (Winkler et al., 2010).

Linkage disequilibrium is defined as a non-random association of alleles at different loci (Du et al., 2007; Waples and England, 2011) because the recombination rate differs from that expected if the loci segregated independently. LD is common between alleles at neighboring loci that tend to be inherited together and associated in a segregating population (Du et al., 2007), but can also be associated with selection (Bulmer, 1971). Characterization of LD is used to assess whether two or more populations can be jointly analyzed in genomic studies, because markers in LD in one population may not be in LD in another population (de Roos et al., 2008), and to make meaningful inferences in populations other than the reference population will depend on the persistence of LD phase between the two populations (Dekkers and Hospital, 2002). The LD level in a population is also used for determining the required marker map resolution to be used in a genomic selection program and testing associations based on QTL scans (McKay et al., 2007). The LD of sufficiently large degree that allows the QTL scan is known as the useful LD (Lu et al., 2012).

To establish genomic evaluations in Mexico, it is important to determine whether multiple HO subpopulations exist. This information determines whether it is necessary to stratify the population by production system and if foreign genomic information, especially from Canada and US, would improve accuracy of predicted breeding values. Thus, the objective of this work was to study the genetic composition and differentiation of the Mexican HO population and the relationship between Mexican cattle with those from Canada and the US. The primary source of stratification of the Mexican population considered was the production system in which those cows performed.

## MATERIALS AND METHODS

### ANIMALS, BREEDS AND GENOTYPES

A total of 625 HO and HO like unrelated cows and sires, born on or after 2005 and genotyped with the Illumina BovineSNP50 or BovineLD Bead Chips were used in this analysis. A total of 149 and 303 animals assigned to the Conv and Lowi systems, respectively, and 173 were from the Canadian and US HO populations. In addition, 162 Jersey (JE) and 86 Brown Swiss (BS) sires from Canada and US were included to help determine if crossbred cows were present in the populations. The animals from the Conv system originated from 16 herds in 6 states of Mexico (Aguascalientes, Guanajuato, Estado de México, Querétaro, San Luis Potosí, and Zacatecas) while the animals of the Lowi system were from 21 herds in 4 states (Estado de México, Jalisco, Puebla, and Tlaxcala). From the 6,836 common SNP markers in both the Illumina BovineSNP50 and the BovineLD Bead Chips a total of 6,617 SNP were included in the analysis after quality control. Markers with a minor allele frequency less than 2% and call rate less than 90% were excluded. Individuals with a call rate less than 90% were also excluded. **Table 1** shows the number and frequency of markers per chromosome included in the analysis.

**Table 1:** Number and frequency of single nucleotide polymorphism (SNP) per chromosome included in the analysis.

Chromosome	Number of SNP by chromosome	SNP percentage by chromosome
1	398	6.01
2	349	5.27
3	310	4.68
4	307	4.64
5	304	4.59
6	310	4.68
7	284	4.29
8	299	4.52
9	272	4.11
10	269	4.07
11	285	4.31
12	225	3.40
13	218	3.29
14	226	3.42
15	223	3.37
16	206	3.11
17	195	2.95
18	176	2.66
19	182	2.75
20	204	3.08
21	186	2.81
22	169	2.55
23	152	2.30
24	177	2.67
25	142	2.15
26	145	2.19
27	139	2.10
28	128	1.93
29	137	2.07

### POPULATION COMPOSITION

The PCA proposed by Price et al. (2006) was used in this study because it models ancestry differences along continuous axes of variation. Genotypes of BS, HO, and JE animals were used. Sire country of origin was considered to explain variation among HO populations and for Mexican cattle; the production system was used as classification variable resulting in three distinct HO populations (Lowi, Conv, and CAN + USA).

### GENETIC STRUCTURE OF MEXICAN HO

The ADMIXTURE package (Alexander et al., 2009) was used for this purpose because it implements a fast model-based estimation that assumes that individuals come from an admixed population with contributions from K ancestral populations. Each K population contributes a fraction q_ik_ for each individual i. Only Mexican HO animals were used for this analysis. Once the K ancestral populations were determined, the most common sire’s country of origin of each ancestral population was identified based on pedigree information.

### LINKAGE DISEQUILIBRIUM DIFFERENCES AMONG HO POPULATIONS

The LD, measured as r^2^ for alleles at two loci was calculated as:

(1) $$r^{2}=\frac{D_{ij}^{2}}{p_{1}p_{2}q_{1}q_{2}}$$

Where *D* is the difference between the observed and the expected frequency of two loci, based on population allele frequencies and assuming random assortment and can be estimated directly from the allele frequencies (Waples and England, 2011), p_1_p_2_q_1_ and q_2_ are the observed frequencies of alleles 1, 2 respectively (Hill and Robertson, 1968). The value, r^2^, is considered the most robust measure of LD. Persistence phase of LD was calculated as the Pearson correlation coefficient between the root of r^2^ between populations for the same pair of SNP (Badke et al., 2012).

Quality control, PCA and LD analysis were performed with SVS Golden Helix software (SNP and Variation Suite Manual v7, 2013; *Golden Helix, Inc.*), persistence phase of LD was calculated using SAS 9.2 (SAS Institute, 2009).

## RESULTS

### POPULATION COMPOSITION

**FIGURE 1:**
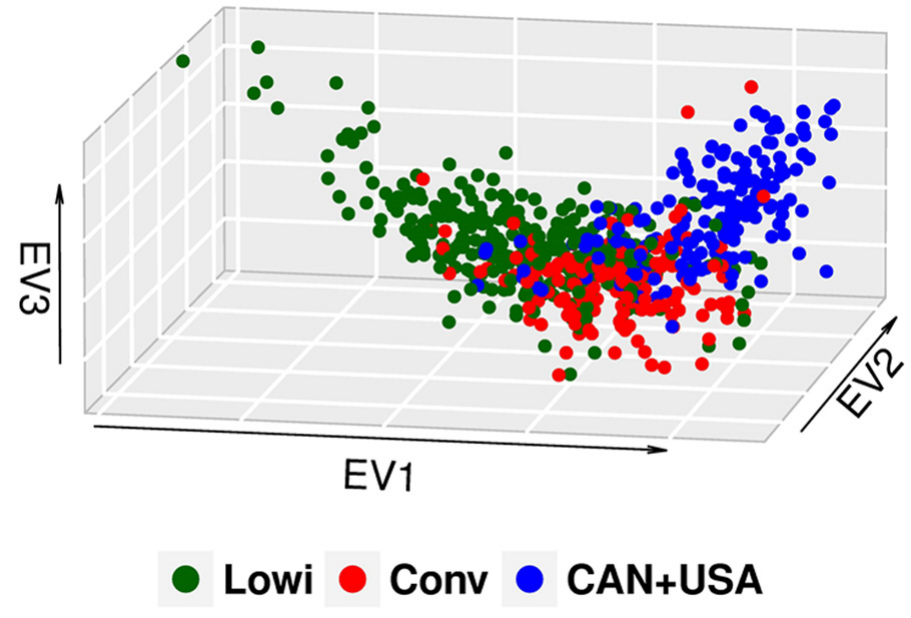
**Principal component analysis (PCA) plot of the North American (CAN + USA) and Mexican Holstein population, classifying the animals according to the production system in which they perform conventional (Conv) and low income (Lowi)**.

**FIGURE 2:**
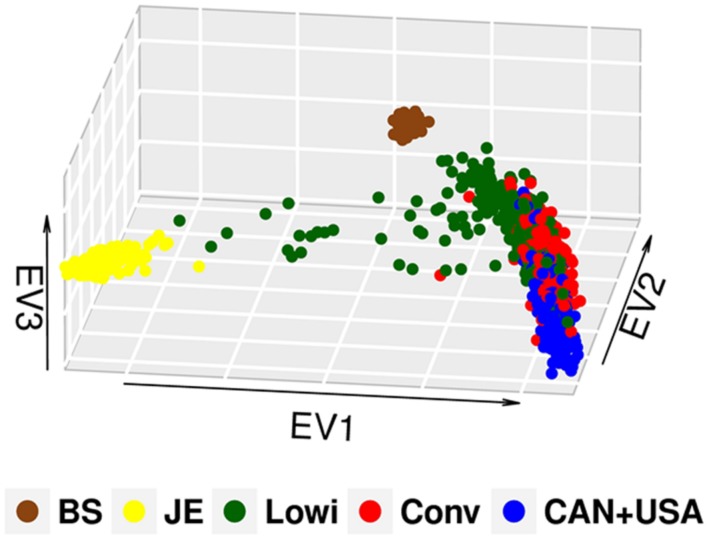
**Principal component analysis plot of the Mexican Holstein cattle of the conventional (Conv) and low income system (Lowi), the North American Holstein (CAN + USA), Jersey (JE) and Brown Swiss (BS)**.

In this study, the first three of 625 components of PCA explained 13% of the observed variation. Population differentiation is observed between the Mexican Lowi and Conv subgroups and the Canadian and US HO cattle (**Figure 1**), despite many common ancestors across these groups. The Conv system seems to be intermediate between the Lowi and CAN + USA groups. Neither Mexican system demonstrated a clustering of animals by country of origin of the sire. PCs for all North American HO cattle along with the JE and BS cattle are shown in **Figure 2**, where the individuals were color coded by breed and population of origin. Crossbred individuals derived from HO and JE or BS were represented by points located between those pure breeds. Crossbreeding was rare in the Conv herds but was much more common in the Lowi systems with a higher proportion of crossing to JE influenced animals than BS.

### GENETIC STRUCTURE OF MEXICAN HO

**FIGURE 3:**
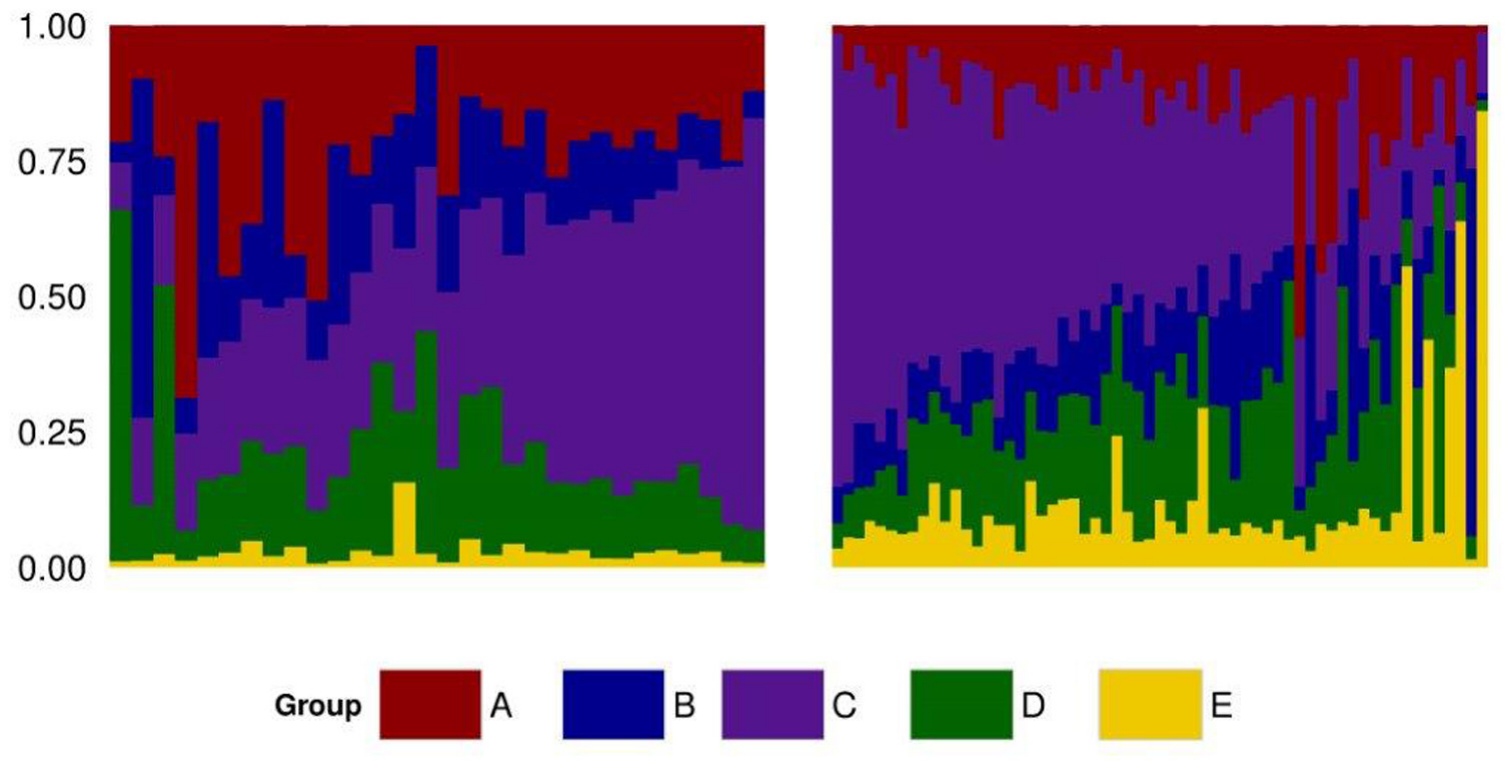
**Admixture plot of the Mexican Holstein population including the conventional (left) and low income system (right), identifying five ancestral groups (A, B, C, D, and E)**.

**FIGURE 4:**
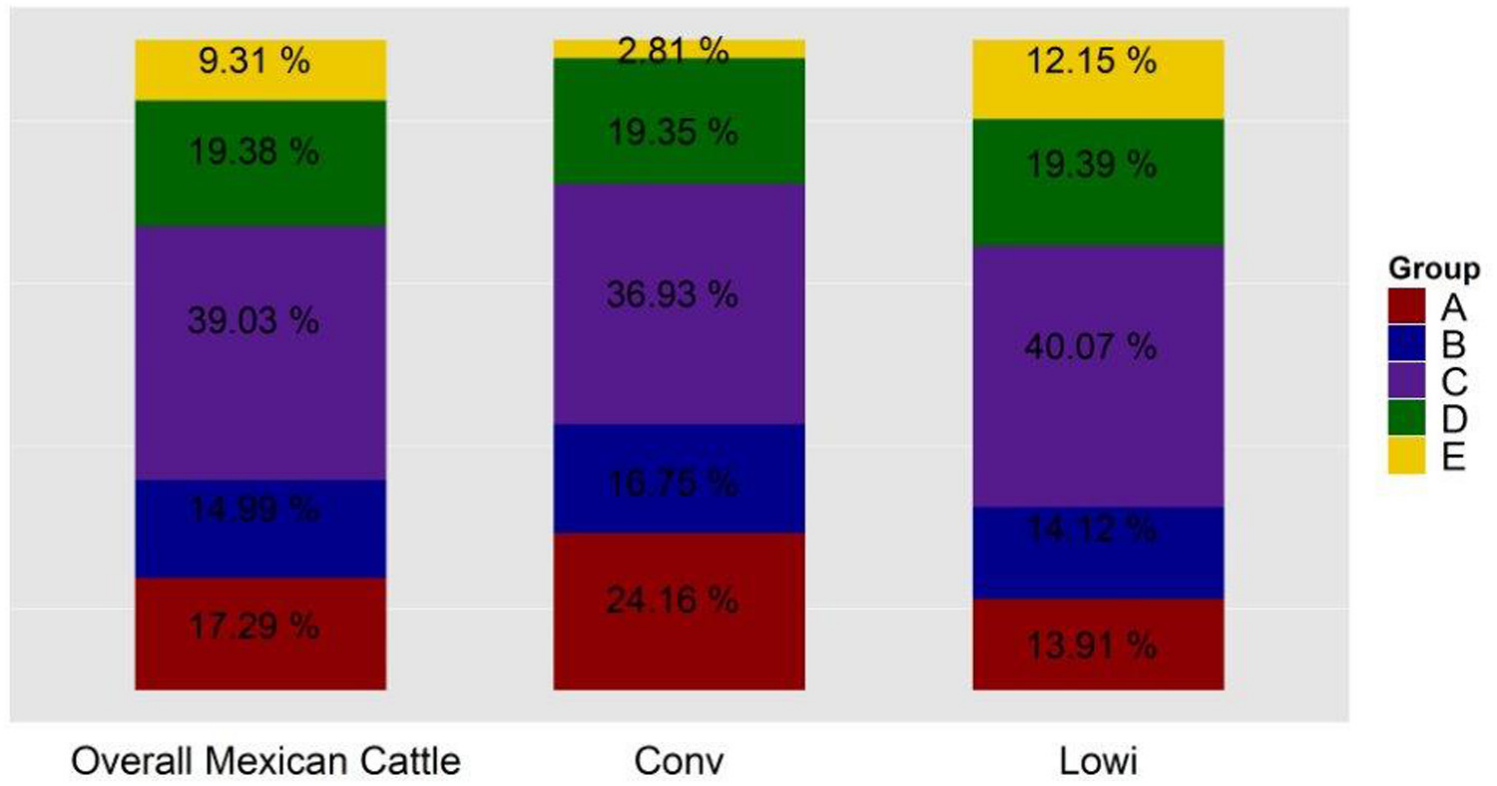
**Genetic composition of the Mexican Holstein population, overall and by the production system in which they perform: conventional (Conv) and low income (Lowi)**.

To identify the genetic structure of the Mexican HO systems and its dependency on the Canadian and US populations, an admixture analysis was performed first for the entire Mexican population and then for each sub-population. For both analyses, the value that best explained the stratification of the population was K = 6. Pedigree records of individual Mexican HO identified their sire’s country of origin as Canadian, US, or Mexican, hence providing an indirect measure of the genetic contribution of these countries to Mexican HO. Indeed, the ADMIXTURE strata identified at K = 6 (**Figure 3**) correlated to the sire’s country of origin. Two of the ADMIXTURE strata were directly related to sires originating from Canada and were subsequently combined into group A with the purpose of investigating country of origin influence. Group B consisted of sires most commonly from the United States (USA). The third strata, group C, consisted of sires from both Canada and US. No unique lineages or discernable characteristics were associated with group C. Another stratum was associated to sires registered in the Mexican herd book and assigned as group D. The last stratum included crossbred animals as evidenced by their genetic similarity to BS and JE breeds in the PC analysis and were assigned to group E. Average ancestry contributions, both overall and by production system, can be observed in **Figure 4**. Population structure showed differences in the genetic composition of the Mexican HO production systems where group C had the largest contribution to the Conv and Lowi populations at 37 and 40%, respectively. In the Conv population, group A (24%), D (19%), B (17%) and E (2.8%) followed while for Lowi animals, group C was followed by D (19%), B (14%), A (14%) and the E (13%). Overall, the contributions of the US, Canada/US, and the Mexican sires were relatively similar among the production systems. The primary differences were within the Canadian and crossbred lineages. The Conv population had approximately 1.7 fold greater contribution from Canadian lineage (group A; Conv-24.16% vs. Lowi-13.91%) and the Lowi population had ∼4.3 fold increase in crossbred lineages (group E; Conv-2.81% vs. Lowi-12.51%).

### LINKAGE DISEQUILIBRIUM DIFFERENCES AMONG HO POPULATIONS

**FIGURE 5:**
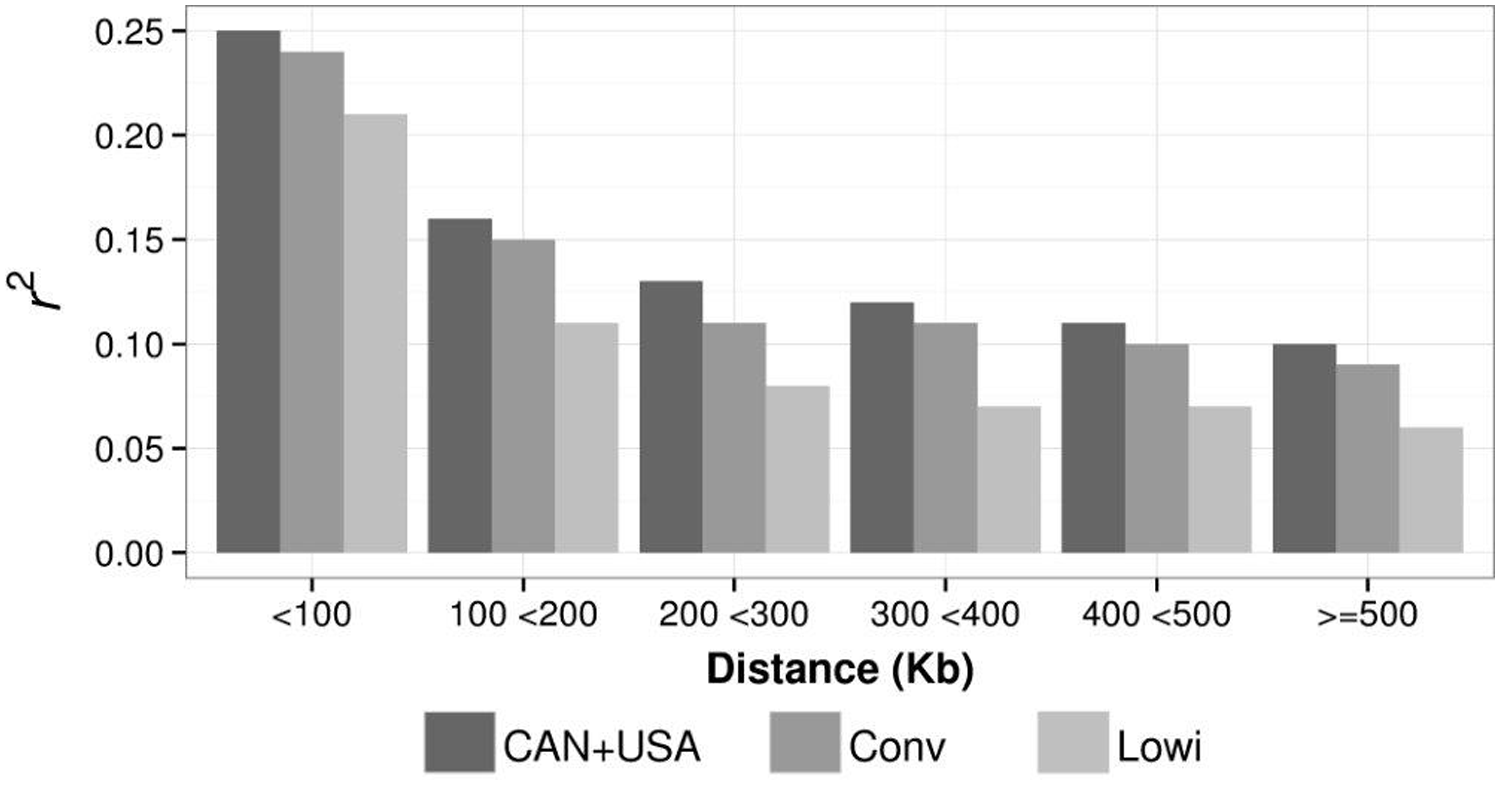
**Linkage Disequilibrium average for different genetic distances between SNP pairs in the Mexican Holstein systems [conventional (Conv) and low income (Lowi)] and the North American Holstein population (CAN + USA)**.

**FIGURE 6:**
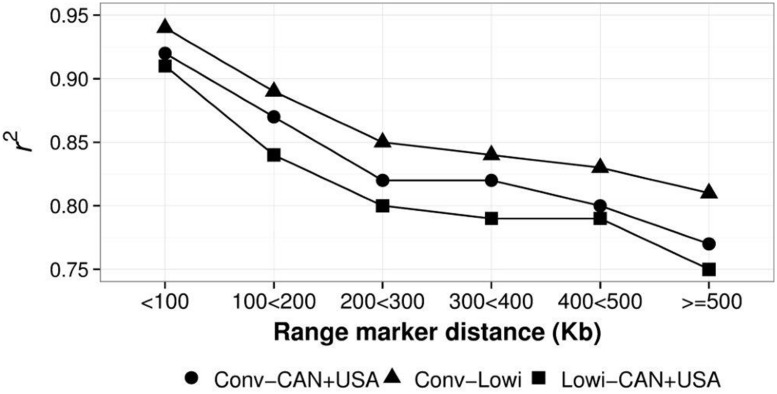
**Persistence of Linkage Disequilibrium phase between North America (CAN + USA) and Mexican Holstein Populations identified as conventional (Conv) and low income (Lowi) at different genetic distances**.

Mean LD, calculated as r^2^ for different distances (at intervals of 100 Kbp) between SNP were calculated for the Conv, Lowi, and CAN + USA populations (**Figure 5**). At all distances, average r^2^ was highest for CAN + USA animals, intermediate for individuals from the Mexican Conv farms, and smallest for cattle representing the Lowi systems. The differences between the CAN + USA and Conv populations r^2^ were quite small (∼0.01) while differences between Conv and Lowi were larger and consistent, ranging from 0.03 to 0.04. The persistence of LD phase between Conv, Lowi, and CAN + USA populations was calculated at the same interval distances as was LD (**Figure 6**). As expected, the persistence of LD phase decreased when the distance between markers was increased. At all intervals, the highest correlations were between Conv and Lowi populations, with the lowest correlation being between Lowi and CAN + USA. At a distance of <100 Kb (with an average of 54.5 kb), the correlations ranged from 0.91 to 0.94 and for a distance >500 kb correlations varied from 0.75 to 0.81 between the Conv-Lowi and Lowi-CAN + USA, respectively.

## DISCUSSION

The results obtained in this study, provide important details to be considered for future genetic research on the Mexican HO cattle and provided the opportunity to measure the genetic relationship of the Mexican animals with the HO populations of Canada and the US.

The Conv system seems to be intermediate in most aspects between the Lowi and CAN + USA, and this population may act as a conduit for germplasm flow from Canada and the US to Mexico.

When the PCA in the HO breed was explored, a difference in the genetic structure was observed between the Mexican production systems and the CAN + USA population, although population overlap suggests that all three groups share common genetic material. In this case the Conv system seems to be a link between the Lowi system and CAN + USA population because breeders of the Conv system provide genetic material (heifers and semen) to the Lowi one and the Conv system has depended genetically from US and Canada for many years (Powell and Wiggans, 1991). The Lowi also obtains genetic material directly from CAN + USA, but at a lower proportion than the Conv one. No specific tendency to group animals for geographic area was found in the Mexican populations, but as in other studies (Novembre and Stephens, 2008), these analyses give us an idea of the genetic background of the population.

The inclusion of JE and BS resulted in individual clusters for both breeds as expected, but more importantly, they helped define the differences between the Lowi and the Conv populations. Results suggest that breeders of the Lowi system occasionally used genetic material of other breeds.

Admixture analysis showed that five different populations, linked to origin country of the sires, comprise the ancestral background of the Mexican HO populations. The two systems showed variation in the average proportion of genetic similarity to the different ancestral populations. The results show a substantial influence of the North American HOs on the Mexican population, and agree with previous results in the Conv system reported by Powell and Wiggans (1991). JE and BS breed influence is visible in both Mexican production systems, but stands out in the Lowi where the use of these dairy breeds seems to be more common. The MEX population was linked through pedigree information to registered Mexican HO bulls related to North American animals. This block also included individuals with unknown sires presumably with similar origins to those of the Mexican sires.

In line with previous studies, decay of LD was observed in this study when the distance between markers was increased (**Figure 5**; Du et al., 2007; de Roos et al., 2008; Sargolzaei et al., 2008; Badke et al., 2012). In general, average r^2^ estimates for the populations in this study were in the range of those reported in other HO populations (McKay et al., 2007; de Roos et al., 2008; Sargolzaei et al., 2008; Qanbari et al., 2010; Zhou et al., 2013), although r^2^ averages for the CAN + USA are slightly higher than those reported for the same population in a group of animals born after 1990 at similar distances between markers (Sargolzaei et al., 2008), except when the distance is less than 100 Kb. Note than the LD of the CAN + USA were higher than the Mexican systems at all compared genetic distances and those of the CONV are slightly higher than those of Lowi. The lowest r^2^ values among the populations were for the Lowi. These lower values may be the result of breeders in the Lowi system introducing other breeds through crossbreeding and incorporating gene migration and genetic drift. This practice may explain the reduction in LD (Lynch and Walsh, 1998). The r^2^ averages for the Lowi decreased rapidly when the distance increased from <100 to 100 and <200 Kb then decreased slowly at distances >200 Kb.

A practical application of LD is determining the number of markers necessary to perform genome wide associations studies (Gautier et al., 2007; McKay et al., 2007; de Roos et al., 2008) at a useful LD (r^2^ ≥ 0.20; de Roos et al., 2008). The useful LD of 54.5 kb found in this study, suggests that at least 53,000 markers should be used to perform genomic analysis within this population. Similar numbers of markers were suggested for other cattle populations (McKay et al., 2007; de Roos et al., 2008).

Because the persistence phase or correlation of r among populations show the genetic relationship between them (Badke et al., 2012), it was used as a measure among the HO populations included in this analysis. Results shown in **Figure 6** confirm the results of PCA, because the higher persistence phase was reported between the Conv and Lowi, followed by the one between Conv and CAN + USA ending with confirmation of the lowest relationship between Lowi and CAN + USA. As it was also reported in other studies (de Roos et al., 2008; Badke et al., 2012) the correlation of *r* among all populations decreased rapidly when the distance between markers increased. The difference between persistence phases among the Conv, Lowi, and CAN + USA ranged from 0.01 to 0.04, lower than that present in other breeds like Angus, Charolais, and JE (de Roos et al., 2008; Lu et al., 2012) or species like pigs (Badke et al., 2012). At distances <100 Kb, the persistence phase between the Conv and Lowi, Conv and CAN + USA, and CAN + USA and Lowi were lower than that reported between Chinese and Nordic HO cattle (0.97; Zhou et al., 2013) and at all measured intervals, similar values were found between Dutch black and white and Dutch red and white HO Friesian bulls and lower values were reported for Australian bulls and New Zealand Friesian cows (de Roos et al., 2008).

Results showed that the US and Canadian and the Mexican HO cattle of the Conv and Lowi have different genetic structures although these populations share much common ancestry. The main difference between the Mexican HO systems is the result of crossbreeding with other breeds, especially in the Lowi system. If joint genomic studies are to be performed between these populations, stratification of populations is recommended. Joint genetic improvement programs of HO animals across North America, i.e., including Mexico, may be established as these populations share genetic material. The useful LD founded in this populations, will determine the minimum number of SNP markers need if joint genomic studies are to be performed.

The considerable similarity between the Conv subgroup with US and Canadian populations means that integration of these groups would be straightforward and should be considered.

## AUTHOR CONTRIBUTIONS

The authors have made the following declarations about their contributions: Conceived and designed the experiments Adriana García-Ruiz, Felipe de J. Ruiz-López, Curtis P. Van Tassell, and Hugo H. Montaldo. Performed experiments and analyze data: Adriana García-Ruiz Data acquisition and interpretation: Adriana García-Ruiz, Felipe de J. Ruiz-López, Curtis P. Van Tassell, Hugo H. Montaldo, and Heather J. Huson. Wrote the paper: Adriana García-Ruiz. All authors approve the manuscript final version.

## Conflict of Interest Statement

The Review Editor Ikhide G. Imumorin declares that, despite being affiliated with the same institute as the author Heather J. Huson, the review process was handled objectively. The authors declare that the research was conducted in the absence of any commercial or financial relationships that could be construed as a potential conflict of interest.
